# Factors Affecting Entrustment and Autonomy in Emergency Medicine: “How much rope do I give them?”

**DOI:** 10.5811/westjem.2018.10.39843

**Published:** 2018-11-13

**Authors:** Sally A. Santen, Margaret S. Wolff, Katie Saxon, Nadia Juneja, Benjamin Bassin

**Affiliations:** * Virginia Commonwealth University School of Medicine, Department of Emergency Medicine, Richmond, Virginia; † University of Michigan Medical School, Department of Emergency Medicine, Ann Arbor, Michigan; ‡ Colorado Permanente Medical Group, Department of Emergency Medicine, Denver, Colorado; § East Central Iowa Acute Care, Department of Emergency Medicine, Cedar Rapids, Iowa

## Abstract

**Introduction:**

During residency, the faculty’s role is to provide supervision while granting the trainee autonomy. This concept is termed entrustment. The goal is appropriate progression from supervision to autonomy while decreasing oversight as residents train. The objective of this study was to better understand the factors affecting the degree of autonomy or supervision faculty choose to provide residents.

**Methods:**

This was a qualitative study of resident and faculty perceptions. We conducted two faculty and two resident focus groups. We then transcribed the transcripts of the audiotaped discussions and coded them using grounded theory.

**Results:**

Analysis of the transcripts yielded four major factors affecting entrustment of residents.

Patient Factors included the acuity of the patient, sociomedical issues of patient/family, and complexity of risk with patient or procedure. For example, “sometimes there are families and patients who are exceedingly difficult that immediately sort of force me [to allow less autonomy].”

*Environmental Factors* included patient volume and systems protocols (i.e., trauma). “If you’re very busy and you have a resident that you already trust, you will give them more rope because you’re trying to juggle more balls.”

*Resident Factors* included the year of training, resident performance, clinical direct observation, and patient presentations. “But if you have a resident that you do not trust […] I tell them you’re going to do this, this, this, this, this.”

*Faculty Factors* included confidence in his/her own practice, risk-averse attitude, degree of ownership of the patient, commitment to education, and personality (e.g., micro-manager). Significant variability in entrustment by faculty existed, from being “micromanagers” to not seeing the patients. One resident noted: “There are some attendings, no matter how much they like you and how much you’ve worked with them, they’re always going to be in your face in the trauma bay. And there’s some attendings that are going to be ghosts.”

**Conclusion:**

Multiple factors affect the amount of autonomy and entrustment given to residents and their level of supervision by faculty, leading to wide variability in entrustment. In the end, regardless of resident, patient, or environment, some faculty are more likely to entrust than others.

## INTRODUCTION

The day residents graduate, they begin independent practice. However, in residency there is wide variability in the amount of autonomy they are given. For faculty, deciding when a resident is ready for unsupervised patient care is not easy. Inappropriate, unsupervised patient care can risk patient safety and increase liability.^1^,^2^ In contrast, a lack of autonomy will impede the resident’s learning and progress toward independent practice.^3^ Faculty need to both entrust residents to practice autonomously, while ensuring safe care. This crucial decision should be founded on the assessment of the resident’s competence in managing the specific task and patient.^4^

Patient care is complex. The collaborative process of patient care between a trainee and an attending is a series of usually tacit decisions on the part of the attending to trust the trainee’s contribution to the patient’s care. The level of trust may be to obtain data such as patient allergies as well as to communicate with patients, accurately interpret diagnostic results, make diagnostic/therapeutic decisions, and perform procedures,^5^ in other words, entrusting the trainee to care for patients autonomously.

The underlying foundation of medical training is that residents receive appropriate, graded responsibility with decreased supervision leading to independent practice when they graduate.^6^,^7^ To complement the Accreditation Council for Graduate Medical Education (ACGME) assessment of competencies, ten Cate and others have proposed entrustable professional activities (EPA).^8^ EPAs are professional tasks that trainees need to master and that require entrustment decisions by clinical supervisors. Degrees of supervision range from 1) the trainee being limited to observing due to limited knowledge or an inability to act to the trainee acting 2) under direct supervision, 3) under indirect supervision or supervision as needed, 4) acting independently, and ultimately 5) supervising others.^9^ As training programs develop and implement competencies such as the ACGME Milestones, ensuring that residents are ready for independent practice is key; understanding entrustment and autonomy will help to facilitate the process to independent practice.^10^

While medical educators advocate entrustment as an assessment decision, the problem lies in how much autonomy is granted to the resident or in other words: “How much rope do we give residents before they hang themselves?” The amount of autonomy and entrustment allowed is a dynamic and fluid process, with a variety of influencing factors. Sterkenburg and ten Cate first examined entrustment by anesthesia faculty in the Netherlands and found that there were four factors affecting entrustment: nature of the task (patient), supervisor, trainee, and circumstances.^11^ This study brought into the foreground the complex process of entrustment.

**Population Health Research Capsule**

What do we already know about this issue?

*Entrustment is the process where facutly vary degrees of supervision while granting the trainee autonomy*.

What was the research question?

*The objective of this study was to better understand the factors affecting the degree of autonomy or supervision faculty allow residents*.

What was the major finding of the study?

*There are four factors affecting entrustment: the resident (e.g., ability), the patient or family (e.g., acuity), the environment (e.g., business of emergency department) and the faculty (e.g., micromanager)*.

How does this improve population health?

*Training excellent emergency physicians to provide care is an important role of residency. To achieve this goal faculty need to progressively allow residents autonomy so that they are safe for independent practice*.

This initial study was conducted in the Netherlands; however, the medical, legal, and insurance environment is different in the United States (U.S.). Medicare and commercial insurers mandate confirmation of medical care by attendings, and with the occasional litigious environment there may be less room for entrustment. This study sought to build on the work of Sterkenburg and ten Cate to investigate the factors affecting how much entrustment or autonomy faculty give residents in a U.S. emergency department (ED).

## METHODS

This was a qualitative study investigating the factors that affect entrustment.^11^ The setting was a four-year, academic emergency medicine (EM) residency program. Residents work in three settings: a public university tertiary-care center; an under-resourced, inner city hospital; and a community ED. In each setting, the faculty is present at all times. The study was determined to be exempt by the university’s institutional review board.

Our study team included two residents and two faculty members to enable us to represent and understand both experiences. We invited all residents and faculty to participate in a focus group and informed all participants about the process. To assure confidentiality and provide an open environment for the residents, we conducted separate focus groups of five to eight people, two for residents and two for faculty. The focus groups were facilitated by the respective study team members; i.e., the residents (NJ and KS) led the resident group. Participants were each given a $15 gift card for their involvement.

The focus groups were semi-structured, audiotaped, and lasted about 60 minutes. As part of the semi-structured questions we used 1 – 3 case vignettes as triggers to explore factors that influence entrustment decisions.^11^ In addition to the case vignettes, we presented to the faculty focus group a trio of residents’ names and asked them how their entrustment would vary based on these residents – specifically, what factors would make them trust one resident over another? The residents were given faculty trios and asked how these individuals managed resident autonomy differently. Our purpose in using names was to ground the discussion in real experiences using specific examples.

Each of the focus group conversations was transcribed and coded without names of participants or the people named in the discussion. Each participant was assigned a number and letter (A or B) depending on their focus group. The data were analyzed using grounded theory^12^,^13^ and informed by the literature on entrustment. Using the constant comparative method of analysis and grouping of data chunks, we recorded emergent themes and refined them after each batch of coding. In contrast to most quantitative research, grounded theory is inductive; the data are used to form the theory by pulling out themes from the focus-group narratives. The faculty and resident teams were coded together to facilitate discussion and deepen understanding of perspectives using Nvivo (QSRv11). Our local and nationally-presented workshops on entrustment with faculty and trainees have served as a member check.

## RESULTS

Four themes emerged with regard to the factors that affect the decision to entrust: the faculty’s underlying disposition toward entrustment; the resident; the patient/family; and the environment. Each of these had specific dimensions (Table).

### Resident Factors Affecting Entrustment

Multiple resident characteristics affected entrustment, including *resident performance*. Faculty observe performance both directly and through patient presentations and then determine how much autonomy the resident should have based on these factors. Lack of ability to orally present a patient is interpreted as poor clinical judgment and grounds for more intensive supervision. In contrast, when a resident is assertive and confident in the initial care of a patient, faculty are more likely to entrust.

“And it’s all, to my mind, dictated by are they demonstrating the ability to take care of the patient with what the patient needs at the moment. And I would step in at any point in time if either they were doing something unsafe or they were missing something that the patient needed, or the patient’s status changed and they failed to recognize it. But so long as they continued to care for the patient appropriately, I would stand there quietly and watch if I had the time to do that.” (faculty 3A)

Another characteristic is the faculty’s *familiarity with and preconceived view* of the resident, which affects their trust. This may be based on working with the resident in the past or by concerns raised in resident assessment meetings. Knowing the resident well will lead the faculty to supervise less or more depending on the circumstances.

“One stands out as having made more mistakes in the past that I personally experienced, and so that one I’m going to supervise much more. And then like [R1] I don’t really know – I haven’t worked with him that much so I have less experience so I’m probably going to [do] more hovering over him.” (faculty 1A)

“I think there’s a lot of preconceived expectations for, you know, this is somebody we know, we’ve worked with them before, we’ve sat in faculty meetings, we’ve evaluated them together, we know what other people think about them as well; so that flavors my experience.” (faculty 6B)

In the absence of specific knowledge of a resident, yet another characteristic includes the *level of training* of the resident. Faculty may intentionally vary their entrustment based on the resident’s post-graduate year. If faculty do not know the resident well, the amount of entrustment may be granted based on performance expectations for the resident’s level of training.

“So, if it was a One [first-year resident], I’d follow them into the room and watch what they were doing. As long as the patient’s vital signs were appropriate I would basically watch them do their assessment, get their history. Once they start to deviate or waste time, or if the patient’s vital signs change, I would jump in. But I would trust them to start the history, start the physical exam, talk to the nurse, you know, get an IV, that sort of thing. A Two [second-year resident], if I knew about it, you know, came overhead, I’d walk in, watch them, same kind of thing, but hopefully they could go further than the One. A Three [third-year resident] I’d probably walk over, vital signs are okay, everything looks fine, I’d go back to whatever I was doing and catch up with them. Same thing with a Four [fourth-year resident].” (faculty 2A)

### Patient

Several patient factors affect the autonomy given to residents. The primary characteristic is based on the *acuity of the patient*. If the patient is acutely ill, faculty are more likely to step in and take control of the care of the patient. On the other hand, if patients are not sick, the faculty will allow more resident autonomy. Similarly, patients in need of *high-risk procedures* such as intubation will require more supervision. *Socially complex patients* such as those with overly concerned parents, the potential for complaints from patient or family, or the need for end-of-life discussions can decrease the amount of autonomy allowed.

“Let’s say a child comes, let’s say it’s an end-stage, end-of-life kind of issue that is horrific and just being sorted out at the moment; that’s just not the time for a trainee, whether he’s seasoned or not, to make a misstep.” (faculty 5A)

“If an attending perceives that there could be a complaint or a problem coming from the family they may not give you as much leeway because they’re afraid of litigation or complaint or other things.” (resident 5A)

### Environment and System

The environment of the clinical setting can have significant effect on the entrustment of trainees. Factors include how busy the department is, the skills or experience of the nursing staff, whether there are systems factors that affect entrustment, and the culture. When the volume and acuity of patients is high and the department is *busy*, for some faculty it means that they allow more autonomy while others become more directive.

“I think that when the department is really busy […] sometimes you have to send somebody in to go do something that you can’t stand there and hold their hand about.” (faculty 5A)

“If you’re very busy and you have a resident that you already trust, you will give them more rope because you’re trying to juggle more balls. But if you have a resident that you do not trust, you’re going to do what (faculty 4A) basically said, I tell them you’re going to do this, this, this, this, this, because I’ve gone [in], I’ve checked the patient and I know what needs to be done, and I don’t have time to have you mucking around because I need you to do these things and report back to me when you’ve completed them.” (faculty 2A)

There are also *system factors* that affect entrustment. For example, patients presenting with potentially life-threatening disorders such as trauma, stroke, and acute myocardial infarction necessitate near-immediate involvement of consultants and diminish the amount of time that faculty can allow the resident to make his or her own decisions. Another systems factor is *nursing capability*. Sometimes while the faculty might not completely trust the resident, they trust an experienced nurse. In the ED, nurses frequently contribute significantly to the care and monitoring of patients. “If [name of nurse] is in the room with a sick patient I’m going to supervise more than if a good nurse is in the room. Because I know that the nurse is going to come get me if the resident does something stupid.”

Finally, in this study the trainees worked at three sites. They noted different *cultures of supervision* between the university setting and increased autonomy and sometimes-minimal supervision at the inner city, under-resourced site.

### Faculty Personality and Approach

As entrustment is a faculty behavior, the final common pathway for the amount of entrustment given to residents was based on faculty factors. Faculty appeared to have a certain approach to entrustment that ranged from those who tended toward a micromanaging supervisory style to those who barely interacted with the residents’ patients, allowing for complete autonomy.

“There’s a general risk tolerance. You could probably put people on a curve and there’s a certain amount of risk that people are willing to tolerate, or I guess like deviation from the plan. And some people are just more relaxed about letting things happen, that … are out of their control and that probably dictates how much autonomy they give to people in general. Even like the same resident will experience different levels of autonomy when they’re with different attendings right across the board, and they can probably predict that even across cases that they’ll get different levels of autonomy because some people will be called micromanagers and some people are just like more laissez-faire about things.” (faculty 1B)

The origin of faculty approach is complex. At times faculty attributed it to their own *risk aversion, lack of comfort with their own skills*, or *their experience as an attending*.

“Especially in a situation where I know that I can probably get them out of it almost no matter what they do, to let them back it up, let them try again, let them talk through it, that sort of thing.” (faculty 4B).

“So, I think my last miss probably plays a huge role in how I let someone work something out.” (faculty 4B)

“I think some of it is their own confidence level, especially at [ ]. One physician in particular that we/I don’t feel has much trust in their own capabilities and so they kind of cast a wide net, want a lot of consults brought in, want every test to be done just because they aren’t confident in their own self. And I think a couple of the others that are very, very conservative, like one in particular, has said he’s been burnt so many times he just doesn’t want to have it happen to him again, and so he knows that he overdoes everything and he will admit that, but he won’t change anything about that. So, again, I don’t know what that is in his personality; maybe it’s a bit of a stubbornness.” (resident 1A)

In contrast, some faculty allow significant autonomy in patient care. At times this is due to a commitment to education, while at other times it is a “laissez-faire” attitude.

“So, I think the independent resident experience is valuable and I think, to get on the soapbox, the way that we supervise everybody now has impacted the degree of training or the quality of training that our current residents get. For that reason, I try and give them as much autonomy and free rein as they need.” (faculty 5B)

“He doesn’t want to know information; he just wants you to take care of the patient and him not see it.” (resident 3B)

## DISCUSSION

We found four themes regarding the factors that affect the autonomy and entrustment of residents: resident, patient, environment, and faculty. While these themes are similar to those noted by Sterkenberg, the manner in which they manifest in EM is different than in anesthesia due to the differences in the environmental context. Since faculty are always present in the ED and patients may be acutely ill or quite stable, there are a series of entrustment or supervision decisions on the part of the faculty. In contrast to Sterkenberg who framed the factors as equal,^11^ we found three of the factors (resident, patient and environment/system) were channeled through the faculty (Figure) as a final common pathway. This means that different attendings choose to allow more or less autonomy regardless of resident and acuity of patient, etc. Where some faculty are comfortable entrusting a lower-level resident with a very sick patient, some faculty will not even trust a fourth-year resident with a less-sick patient. Entrustment decisions are a series of dynamic decisions made by the faculty based on the three factors of resident, patient, and environment.

While this is not the first study of entrustment in EM,^14^,^15^,we believe that our efforts contribute to a deeper understanding of the factors involved in entrustment decisions, particularly of the faculty factor. Given our findings of the strong role of faculty personality and approach, future work will be needed to determine how an individual faculty’s predilection for entrustment affects their entrustment decisions.

## LIMITATIONS

This study was an initial step toward understanding entrustment in the ED. However, there are limitations implicit in our qualitative methods. Qualitative studies are descriptive and are not intended to test inferences about causation or associations. Respondents may have felt a need to provide answers showing a social desirability toward entrustment. We conducted four focus groups, but it is possible that we might have found more or different subthemes if we had continued with more focus groups or if we had combined residents and faculty in the same groups. To control for power dynamics, we chose to keep residents and faculty separate. In addition, because participants were recruited from a single program, generalizability is limited.

## CONCLUSION

Important factors affect the amount of autonomy and entrustment that faculty give to residents and the level of supervision residents get from faculty, leading to wide variability in entrustment. The four key factors are resident, patient, environment, and faculty. In the end, regardless of resident, patient, or environment, some faculty are more likely to entrust than others.

## Figures and Tables

**Figure:**
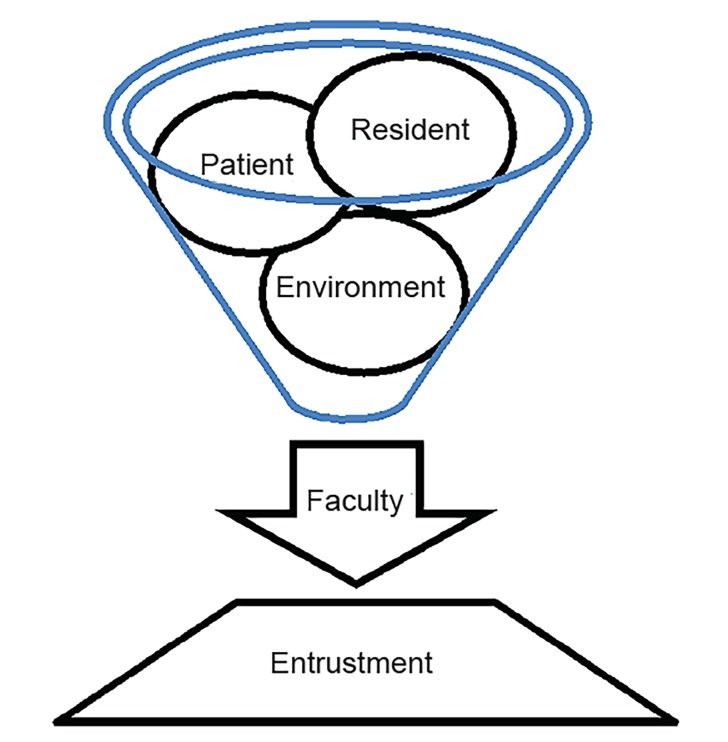
Dynamic relationship of entrustment.

**Table:** Themes and subthemes of factors affecting faculty entrustment of patient care to residents.

Resident
Performance
Oral presentation/plan or overview of the case
Familiarity and preconceived view of the resident
Level of training
Resident’s apparent self-confidence
Environment
How busy was department
Systems factors - (e.g., stroke alert requires faculty presence)
Nursing capability
Culture of supervision
Faculty
Personality and approach
Comfort with own skills/experience
Disposition to micromanage
Risk averse
Sense of medical responsibility vs. educational responsibility
Patient/family
Acuity/severity
Difficulty of problem or task
Risk to patient (procedures)
Socially complex patients and family issues
